# Polymorphisms at Myostatin Gene (*MSTN*) and the Associations with Sport Performances in Anglo-Arabian Racehorses

**DOI:** 10.3390/ani11040964

**Published:** 2021-03-30

**Authors:** Emanuela Pira, Giuseppe Massimo Vacca, Maria Luisa Dettori, Gianpiera Piras, Massimiliano Moro, Pietro Paschino, Michele Pazzola

**Affiliations:** 1 Local Health Authority 7 Pedemontana, Via dei Lotti 40, 36061 Bassano del Grappa, Italy; emanuelapira@hotmail.com; 2 Department of Veterinary Medicine, University of Sassari, Via Vienna 2, 07100 Sassari, Italy; gmvacca@uniss.it (G.M.V.); mldettori@uniss.it (M.L.D.); ppaschino@uniss.it (P.P.); 3 Local Health Authority of Oristano, Via Carducci 35, 09170 Oristano, Italy; gppiras@hotmail.com; 4 Local Health Authority of Nuoro, Via Amerigo Demurtas 1, 08110 Nuoro, Italy; massimilianomoro@virgilio.it

**Keywords:** myostatin, racehorse, Anglo-Arabian horse breed, non-genetic effect

## Abstract

**Simple Summary:**

Myostatin is a protein which plays a key role in the regulation and differentiation of cells. The encoding gene, *MSTN*, and its mutations have been studied in cattle because of the association with increased muscle masses. In horses, some polymorphisms at *MSTN* have favorable effects on sport performance, as evidenced for the Thoroughbred breed. The Anglo-Arabian horse is a very common breed in many Mediterranean countries because of its extreme adaptability, but genetic investigations are rare in the literature. The present study evidenced the relatively high variability of this breed at *MSTN*. Many phenotypic effects, such as sex and genealogy, affected the results but, overall, the same polymorphic sites already reported for Thoroughbred horses affected race performances also in Anglo-Arabians.

**Abstract:**

One hundred and eighty Anglo-Arabian horses running 1239 races were sampled for the present study. DNA was extracted from the blood and myostatin gene, *MSTN*, was genotyped. Moreover, prizes won and places were achieved for the 1239 races to perform association analyses between the different genotypes and sport traits. Two SNPs already reported in previous studies regarding the Thoroughbred breed, rs69472472 and rs397152648, were revealed as polymorphic. The linkage disequilibrium analysis investigating the haplotype structure of *MSTN* did not evidence any association block. Polymorphism at SNP rs397152648, previously known as g.66493737 T>C, significantly influenced sport traits, with heterozygous horses TC showing better results than homozygotes TT. The portion of variance due to the random effect of the individual animal, and the other phenotypic effects of sex, percentage of Arabian blood and race distance, computed together with the genotype at *MSTN* in the statistical models, exerted a significant influence. Hence, this information is useful to improve knowledge of the genetic profile of Anglo-Arabian horses and a possible selection for better sport performance.

## 1. Introduction

Myostatin, also known as growth/differentiation factor-8 (GDF-8), is a protein belonging to the superfamily of signal molecules called transforming growth factors beta (TGF-β). These are largely expressed during embryonic development, play a role in regulation and homeostasis of various tissues in adults [1] and affect many other functions such as cell differentiation, reproduction, bone morphogenesis and wound healing [2]. Myostatin, under physiological conditions, controls skeletal muscle development and reduces the muscle size: mouse embryos lacking the myostatin gene develop hyperplasia, due to an increased number of muscle cells, and, after birth, hypertrophy due to a high increase in the size of each fibre [3].

The encoding gene *MSTN*, as the other members of the TGF-β family, is highly conserved among species and homologous sequences are found in mammals as well as in chickens [4]. In livestock species, particular interest has been paid to the myostatin [5] and the mutations at *MSTN*, since the discovery of its key roles in the abnormal increase of muscle masses are found in some beef cattle breeds. Genetic studies have revealed that nucleotide variations at the *MSTN* gene are responsible for the phenotype known as double muscle in Belgian White and Blue cattle [6,7] and in other breeds, such as the Piedmontese [8].

In the horse species, some breeds as like as the Thoroughbred breeds has been selected for centuries to improve the race performance and became athletic horses [9]. Although the athletic phenotypes are markedly influenced by environmental factors such as breeding, management and training, these are also under the control of genetic factors [10]. Nevertheless, there are still a few studies focusing on genetic regulation of horses’ sport performance [11,12] and a few candidate genes have been identified [13,14]. The *MSTN* gene is located on Equus caballus (horse) chromosome 18, Accession number NC_009161.3 (66605150..66610122, complement), on assembly EquCab3.0 (GCF_002863925.1). It is about 4972 bp long, spans three exons and two introns, and encodes a 375 aminoacids precursor protein, containing a signal sequence, a propeptide amino-terminal domain and a carboxy-terminal domain, which is the active form of the molecule [3]. Genome wide association studies (GWAS) have identified one SNP (single nucleotide polymorphism) at horse *MSTN*, rs397152648 (previously identified as g.66493737 T/C), as predictive of genetic potential, because of its association with the best race distance in Thoroughbreds [15]. It has been demonstrated that rs397152648 C/C horses have best performances in short-distance races, T/C horses perform better in middle-distance races, and T/T horses in stamina races [15]. Studies on sport performance regarding Thoroughbred horses have demonstrated that, for the SNP rs397152648, the T allele was ancestral, while the C allele was later introduced from a single event during the breed formation, and later transmitted to most of the offspring by the lineage of the two stallions Nearctic and his son Northern Dancer [16]. Other sport and draught horse breeds have been later investigated at *MSTN* [17,18]. *MSTN* has been significantly associated also with precocity traits [19] and the results regarding the influence on sport performances have been confirmed and strengthened in a very large population of Thoroughbred horses, using high-density SNP arrays [20]. On the other hand, the continuous research in the field of genetic improvement of animal production, in this case sport performance of horses, is not free of criticism because the possible effects of gene doping on the health are substantial [21].

The Anglo-Arabian horse is a very common breed in many Mediterranean countries. Many breeders and their stables are located in France and Italy, with a particular and appreciated nucleus in the island of Sardinia (Italy) where, over the last two centuries, Thoroughbred and Arabian stallions were crossbred with local mares [22,23,24]. Nowadays, this breed is reared in many other countries and, since the 1990s, all the new-born foals are registered in the stud books under the common requirements of the International Confederation of the Anglo-Arabian horse breed [25]. Because of its extreme adaptability, it is used for many equestrian disciplines in both the categories of jump and race sports. The phylogenetic relationships of Anglo-Arabian horses with Arabian and other Italian horses, based on mitochondrial DNA analyses, were recently investigated [22,24] while, to our knowledge, there is neither a survey in the literature assessing the variability of the myostatin gene nor a study evaluating association between *MSTN* variants and race performance traits in Anglo-Arabian horses.

The objective of this study was to analyse the *MSTN* gene sequence and evaluate its variability and association with phenotypic sport performance traits in the Anglo-Arabian horse breed.

## 2. Materials and Methods

### 2.1. Animals and Phenotypic Data

Ethical review and approval were waived for this study. No specific authorization from an animal ethics committee was required. Blood samples were collected by private and official veterinarians of the local health authorities (ASSL) during clinical samples, control or eradication sanitary programs not linked with the present study. Sampled horses belonged to private breeders, who joined the present study on a voluntary basis.

Data and samples were collected from 180 horses bred by 119 different breeders with stables located in the regional territories of Sardinia and Tuscany, Italy. All the horses were officially registered in the stud book of the Anglo-Arabian breed.

General data of each horse were downloaded via the public online databank of the Italian horse breeds register (http://www.unire.gov.it/index.php/ita/Operatori/Banca-dati accessed on 10 January 2017): name, microchip number, sex, year of birth, breeder, the complete genealogy up to the fourth generation and the percentage of Arabian blood. Data regarding the sporting career of each horse were downloaded via the public online databank of the Italian horse races (http://www.hippoweb.it/prestazioni.php accessed on 10 January 2017): races ran at the age of three years, which is the debut age for Anglo-Arabian racehorses; for each of these races: date, racecourse, distance of the race, number of horses at the starting line, order of arrival at the finish line, purse, price won, name of the jockey, trainer. As regards prizes won before 2002, the online databank automatically provides the exchange rate between the currencies in Italian lira and euro. Data from a total of 1239 races were collected.

To achieve further information from the above-mentioned phenotypes and a deeper vision of sport performances, new parameters were calculated for the individual horse and the race. As regard the individual horse, four new parameters were considered. The prize index, PrIndex, aimed to compute the prize won on the basis of the race ran in career; it was calculated as the ratio between the total prize won and the number of races ran at 3 years; it ranged from 0 and, theoretically, to infinite, and the higher the PrIndex was, the more prominent the result. The place indexes aimed to evidence the places on the basis of the race ran at 3 years; these were two: calculated for the first places, 1st-PlIndex, as the ratio between the number of first places and the number of races ran by that horse, and for total places, Tot-PlIndex, as the ratio between the number of places (sum of first, second and third places) and the number of races ran by that horse; place indexes ranged between 0 and 1, and the higher the indexes were, the more prominent the results. As regards the individual race (only races at the debut age, three years), three new parameters were considered. The arrival index, ArIndex, aimed to differentiate the places on the basis of the competitors; it was calculated as the complement to one of the ratios between the number of horses at the starting line and the place of the horse at the finish line; it ranged between 0 and ~1, and the higher the ArIndex was, the more prominent the result. The purse index, PuIndex, aimed to compute the prestige of the race and differentiate the prizes won on the basis of the total purse; it was calculated as the ratio between the prize won and the purse; it ranged between 0 and 1, and the higher the PrIndex was, the more prominent the result. The success index, SuIndex, aimed to compute both the competitors and the prize won; it was calculated as the multiplication between the ArI and the prize won; it ranged from 0 and, theoretically, to infinite, and the higher the SuIndex was, the more prominent the result.

### 2.2. Blood Samples and Genotyping

Individual blood samples from each horse were collected from the jugular vein using K3EDTA vacuum tubes (Vacutest Kima, Azergrande, PD, Italy) and DNA extracted using the Gentra Puregene Blood Kit (QUIAGEN, Venlo, The Netherlands) according to the manufacture instructions. Based on the *MSTN* gene sequence (NC_009161.3) 10 primer pairs were designed using Primer3Plus software (http://www.bioinformatics.nl/cgibin/primer3plus/primer3plus.cgi/ accessed on 10 January 2017) to amplify by PCR 10 overlapping DNA fragments. After sequencing, the 10 amplified DNA fragments gave information about the entire MSTN sequence, including 600 bp of the promoter and 600 bp of the 3′UTR region (Table S1). Primers were designed to obtain overlapping fragments. For each primer pair, amplification was carried out in a final volume of 25 μL, and the following conditions: 100 ng of genomic DNA; 1.5 mM of MgCl2 (NZYTech, Lisboa, Portugal); 0.2 mM dNTPs; 1×PCR buffer (NZYTech); 0.2 μM of each primer; 1 unit of NZYtaq DNA polymerase (NZYTech) and H_2_O up to the final volume. The amplification programs were performed on Thermal Cycler Mastercycler ep (Eppendorf, Darmstadt, Germany) according to the following scheme: an initial denaturation at 94 °C for 3 min, followed by 35 cycles at 95 °C for 20 s, 56 °C for 30 s (55 °C only for fragment 1) and 72 °C for 50 s, and a final extension at 72 °C for 5 min. The PCR products were purified using the ChargeSwitch^®^ PCR Clean-Up Kit (Invitrogen, Carlsbad, CA, USA) and were sequenced using an Applied Biosystems 3730 DNA Analyzer (Applied Biosystems, Foster City, CA, USA). The entire gene sequence was obtained from two individual horses and the two complete *MSTN* sequences were submitted to GenBank (https://www.ncbi.nlm.nih.gov/genbank accessed on 10 January 2017) and given accession numbers KY746356 (6195 bp long, isolate 4) and KY746357 (6194 bp long, isolate 11). The two primer pairs SHOR3737F-SHOR3737R (DNA fragment 3, intron 1) and SHOR_6F-SHOR_6R (DNA fragment 6, intron 2) were used for genotyping by sequencing the sampled population (Table S1). All the sequences obtained were submitted to GenBank (Accession numbers KY746358–KY746537, intron 1; KY746538–KY746713, intron 2).

### 2.3. Analysis of Genetic and Phenotypic Data

The softwares Phred, Phrap and Crossmatch were used to achieve data quality of sequence plots [26,27], PolyPhred to compare sequences [28,29] and Consed to examine the variation sites evidenced by PolyPhred [30]. The Haploview software [31] was used to calculate allele frequency, observed and expected heterozygosity, and the Hardy–Weinberg equilibrium at each polymorphic site, and linkage disequilibrium (LD) among the detected SNPs.

Phenotypic traits regarding sport performances of the 180 individual horses (total prizes won; prize index, PrIndex; place indexes for first places, 1st-PlIndex, and total places, Tot-PlIndex) were analysed only for the debut year (three years of age) using the general linear model procedure and the following model (M1):

Y_hijk_ = μ + G_h_ + S_i_ + ArB_j_ + e_hijk_ (1)

where Y_hijk_ is the observed trait; μ is the overall intercept of the model; G_h_ is the fixed effect of the hth SNP genotype (h = 2 to 3 levels: the two homozygotes and the heterozygote); S_i_ is the fixed effect of the ith class of sex (i = 2 classes; class 1: male; class 2: female); ArB_j_ is the fixed effect of the jth Arabian blood percentage (j = 2 classes; class 1: <50%; class 2: ≥50%); and e_hijk_ is the random residual ~ N (0, $\sigma_{e}^{2}$), where $\sigma_{e}^{2}$ is the residual variance.

Phenotypic traits regarding sport performances of the 1239 individual races (arrival index, ArIndex; purse index, PuIndex; success index, SuIndex) were analysed only for races ran during the debut year (three years of age) using the mixed model procedure and the following model (M2):

Y_hijklmn_ = μ + G_h_ + S_i_ + ArB_j_ + D_k_ + H_l_ + J_m_+ e_hijklmn_ (2)

where Y_hijklmn_ is the observed trait; μ is the overall intercept of the model; G_h_ is the fixed effect of the hth SNP genotype (h = 2 to 3 levels: the two homozygotes and the heterozygote); S_i_ is the fixed effect of the ith class of sex (i = 2 classes; class 1: male; class 2: female); ArB_j_ is the fixed effect of the jth Arabian blood percent (j = 2 classes; class 1: <50%; class 2: ≥50%); D_k_ is the fixed effect of the kth class of race distance (k = 3 classes; class 1: from 1000 to 1400 m; class 2: 1450–1800 m; class 3: 1850–2400 m); H_l_ is the random effect of the lth individual animal (l = 180 horses), pooling all the possible influence of the individual animal, e.g., genealogy, breeder and trainer; J_m_ is the random effect of the mth jockey (m = 102 jockeys); e_hijklmn_ is the random residual ~ N (0, $\sigma_{e}^{2}$), where $\sigma_{e}^{2}$ is the residual variance.

Both for the individual horse and race, we analysed one sport performance trait for each SNP at a time, only SNPs with minimum allele frequency, MAF, higher than 0.05 (Table 1) and genotypes with frequency higher than 0.05. The effects were declared significant at *p* < 0.05 and multiple comparison of least square means (LSM), for the factors with more than two levels, was performed by using the Bonferroni method at α = 0.05. Statistical analyses were performed using SAS software (SAS version 9.4, SAS Institute Inc., Cary, NC, USA).

## 3. Results

A total of 180 horses were sampled. These horses were born from 1987 to 2013, from 72 different stud farms (from a minimum of 1 to a maximum of 30 horses per stud farm) and 156 mares (from 1 to 3 horses per mare), 119 breeders (from 1 to 8 horses per breeder), trained by 47 trainers (from 1 to 32 horses per trainer). These horses, at the age of three years, which is their debut year, participated in 1239 races (from a minimum of 1 to a maximum of 17 races per horse), ridden by 102 jockeys (from 1 to 143 races per jockey) and ran in ten racecourses (from a minimum of 2 to a maximum of 649 races per racecourse). Table S2 summarizes the effects, levels and sample size investigated in the present study.

### 3.1. MSTN Variation

For the present study, sequencing of the entire horse *MSTN* gene, including about 600 bp downstream and upstream, was performed in two subjects of Anglo-Arabian breed (KY746356 and KY746357). Sequence analysis evidenced 14 different polymorphic sites (Table 1). All the SNPs were in the two intronic regions of the gene (9 in the first intron and 5 in the second), while exons and the upstream and downstream flanking regions did not display any polymorphism. Thirteen out of fourteen nucleotide variations were SNPs (including 8 transitions and 4 transversions), and one variation was an indel, located 40 bp upstream the junction between intron 2 and exon 3 (rs1095048842).

To investigate the possible effects on sport traits of the described nucleotide variations, we performed resequencing of the relevant DNA fragments, including intron 1 and intron 2 (Table S1) in the 180 sampled Anglo Arabian horses. A total of 10 SNPs were genotyped, seven located in intron 1 and three in intron 2. Only four SNPs displayed MAF > 0.05. The SNP rs397152648 T/C (previously reported as g.66493737T>C) was shown to be polymorphic in the present study, with MAF at 0.13. All genotyped SNPs were in the Hardy–Weinberg equilibrium. The LD analysis carried out to investigate the haplotype structure spanning 2313 bp of the *MSTN* gene, did not evidence any association block, but only one possible recombination hotspot within intron 2 (Figure S1).

### 3.2. Association Analysis between MSTN Polymorphisms and Sport Performance Traits

Results of the statistical analysis (raw *p*-values and significance) from models (M1) for the individual horse and (M2) for the race, performed to investigate the influence of SNP genotypes at *MSTN* on sporting performance traits, are reported respectively in Table 2 and Table 3. We analyzed only the four SNPs showing MAF > 0.05 and genotypes with frequency higher than 0.05, together with fixed and random effects.

The genotype effect significantly affected sport performances only for rs397152648, formerly identified as 66493737 by Hill et al. [15] The total prizes won and PrIndex (Table 2), and the two indexes of arrival and success (Table 3) were affected at *p* < 0.05. Among the fixed effects, the percentage of Arabian blood was always significant for the four SNPs and all the traits (Table 2 and Table 3). Horses with values of Arabian blood percentage higher than 50% showed the best values for all the SNPs and indexes (data not shown in tables). The effect of sex significantly affected the total prizes won and PrIndex (Table 2), and purse and success indexes (Table 3) and the best results were obtained by male horses (data not shown in tables). The race distance had a high significant influence (always at *p* < 0.001) on all the indexes computed for the races, with the best results regarding the arrival and purse indexes recorded for the class of races from 1000 to 1400 m, while regarding the success index for races between 1850–2400 m (data not shown in tables). Finally, the random effect of the individual animal represented a portion of variance from a minimum of 10.90 to a maximum 26.53%, while the one due to the jockey was from 1.08 to 3.78% (Table 3).

Least square means and comparison among the different genotypes are reported in Table 4. Homozygotes CC at SNP rs397152648 (3 horses running 18 races), CC at rs1095048831 (2 horses running 16 races), and TT at rs1095048829 (6 horses running 51 races) were not computed because of the genotype frequency lower than 0.05. As regard SNP rs397152648, the only one significantly affecting sporting traits, the heterozygotes TC showed better results than homozygotes TT (Table 4).

## 4. Discussion

The *MSTN* sequences analysed in full in this study included the promoter region of the gene, and we observed that the 227 bp SINE (short interspersed nuclear element) insertion evidenced in the *MSTN* promoter region of Thoroughbred horses, in linkage with SNP rs397152648C genotype [15,32] was not revealed in the sequences of Anglo-Arabian horses of the present study. In addition, the SNPs rs69472469, rs69472470 and rs69472471, and the SNP rs782836148 at the 3′UTR, were reported as polymorphic in the literature [15,18,33,34], were homozygous in the horse *MSTN* gene of this study, with TT, TT, CC and AA genotypes, respectively. The variability observed in the Anglo-Arabian breed of this study may be derived from the fact that Anglo-Arabian horses, in particular the subjects reared in Sardinia, originate from a pool of about three hundred native Sardinian mares, which were initially crossed with Arabian stallions, and later with Thoroughbred and Anglo-Arabian stallions [23,24]. That crossbreeding practices has realistically led to a continuous increase of genetic variability was also confirmed by Giontella et al. [24], who highlighted that the current population of Anglo-Arabian horses has a lower average inbreeding coefficient than other Italian breeds. Unlike the Anglo-Arabian breed, the Thoroughbred is derived from a few ancestors, as is also testified by the local names given in France and Italy, respectively *Pur-sang Anglais* and *Purosangue Inglese*, which stands for English pure blood. Indeed, some authors hypothesize that the low variability in Thoroughbred horses is derived from the high degree of inbreeding [35,36], as the breed is built on a pool of three sires and about 30 mares. Similar results of variability as the present study have been reported by Baron et al. [33], who have sampled 20 different horse breeds, including Thoroughbred, Arabian, German, Portuguese and Spanish breeds. Those authors have evidenced the presence of 10 polymorphic sites only at *MSTN* exon 2, some of those breed-specific, e.g., ECA18:66,492,906 (RefSeq NC_009161.2) 2279A> C for the Arabian breed.

As regards the association analysis performed for the present study, we used only races of the debut year of horses to have the highest level of homogeneity. Different to Thoroughbreds, which begin their racing careers at the age of two, Anglo-Arabians’ debuts take place at the age of three years. During the debut year, horses participate in races which are exclusively limited for three year entrants. Later, several horses are retired from the tracks and there are no more possibilities to compare horses of the same age. The significant influence of the SNP rs397152648 T>C on sport performance evidenced in the Thoroughbred breed has been studied in depth to perform the creation of the “speed gene test” [37,38] and predict the possible career of sport horses via the genetic basis. In the present study, association of SNP rs397152648 and sport performance of Anglo-Arabian horses evidenced that heterozygous horses at SNP rs397152648 (genotype TC) had the best results for four out of the seven analysed performance indexes, when compared to homozygous TT genotypes. Indeed, TC horses won higher total prizes in the debut year of about 3100 €; a higher average prize index (PrIndex) of about 370 €; and showed better arrival index, plus 14%, and purse index, at 20% (differences calculated from data in Table 4). This could be explained by the general positive effect of heterosis on animal productive traits [39,40] and, for the horse species, it has been reported that in French trotters, heterozygotes at one locus of the *DMRT3* gene have better sports performance than homozygotes [41]. Finally, although the finding regarding the effect of SNP rs397152648 and sport performance was statistically significant, caution should be paid because of the relative low frequency of the CC homozygotes.

It is worth noting that the portion of variance due to the random effects of the individual animal was relatively high, while the one due to the jockey was lower. Almost all the phenotypic effects, the so-called “non-genetic influence” [20], computed together with the genotype effect at the *MSTN* SNPs in the statistical models, exerted a significant influence. The effect of sex was significant for two out of four traits regarding the individual horse and two out of three traits regarding the race. The best results, in accordance with the literature, were recorded for males. Two studies about Thoroughbred horses report that males are normally more competitive than females [42] and are more likely to have a longer career [43]. This is attributable to the fact that in females the stress response during races is more accentuated than in males [44,45]. This last result was also achieved in a study including Anglo-Arabian horses reared in Sardinia, which has evidenced that mares participating in a stressful event have higher β-endorphin levels than males and geldings [46].

Many hypotheses could be advanced regarding the results about the supremacy in all the traits for horses with the prevalence of Arabian blood. Based on the findings by Giontella et al. [23], which reported that the higher is the Arabian blood percentage, the smaller are body measures and horse size of Anglo-Arabian horses, we can speculate that the smaller size has supported better sport performance in horses sampled for the present study. Other data which could have influenced this result is attributable to the higher number of races ran by horses with Arabian blood prevalence, which were able to run in the debut year an average number of races 1.2-fold higher than horses with Arabian blood lower than 50% (data in Table S2).

As regard the effect of the race distance, noteworthy influences of sex and the genotype on performance in different race distances is reported in the literature. Harkins et al. [42] have reported that horses are more competitive, in that a study of the males demonstrates that they have the best performance in races with the longest distances. Moreover, Hill et al. [15] have evidenced that CC horses at SNP rs397152648 are more suitable for short-distance races and TT for longer distances. That last result is not easily comparable with our study because of the almost total absence of CC homozygotes in the sampled Anglo-Arabian population and the substantial differences in classes of race distance between the two studies. The *MSTN* gene has a significant impact on biometric traits [47,48] and muscle fibre [49], and the related and pooled effects of *MSTN* genotype, the breed and race distance are well described by Rooney et al. [50] Those last authors consider that TT horses at SNP rs397152648 have a greater body mass and the SINE insertion influences muscle fibre composition and types, with different genotypes, which are consequently best suited for sprint or stamina sport activities. Indeed, the performance in relation to the distance of races has been later confirmed by Dall’Olio et al. [51] and Petersen et al. [52] who have demonstrated the functional role of *MSTN* gene in horse breeds used for sprint races, like the Quarter Horse. Nevertheless, similarly to the study by Hill et al. [15], we tried to investigate which could be the best genotype of Anglo-Arabian horses according to the race distance. Hence, we performed a supplementary statistical analysis similar to model (M2), appending the interaction effect between the genotype at SNP rs397152648 and the classes of race distance but the *p*-values were non-significant (0.917 for ArIndex, 0.501 for PuIndex and 0.915 for SuIndex; model not reported in the paragraph Materials and Methods and data not shown in tables).

## 5. Conclusions

The Anglo-Arabian is a versatile horse breed. The present study aimed to improve the knowledge on this breed and revealed that the *MSTN* locus in the analysed population has a relatively high variability and some SNPs were reported for the first time. With regard to the association analysis of genetic polymorphism with sport indexes, it was confirmed that SNP at ECA18 rs397152648 T>C, already studied in Thoroughbred horses, affected race performances also in Anglo-Arabians. The genetic selection to obtain better performance of horses could be a possibility to be explored, but it is clear that this is not the only way, because the other phenotypic effects studied showed high values of significance.

## Figures and Tables

**Table 1:** SNPs at the equine *MSTN* gene in Anglo-Arabian horses (*n* = 180).

SNP ID	Chr Position	HGVS Name	Gene Region	ObsH	ExpH	HWpv	MAF	Alleles
rs69472472	66609244	c.373+506T>C	intron 1	-	-	-	-	-
rs1095048834	66609160	c.373+590T>G	intron 1	-	-	-	-	-
rs1095048850	66608770	c.374−850G>A	intron 1	0.04	0.04	1.0	0.02	G:(A)
rs1095048833	66608717	c.374−797T>C	intron 1	0.02	0.02	1.0	0.01	T:(C)
rs1095048832	66608687	c.374−767T>C	intron 1	0.02	0.02	1.0	0.01	T:(C)
rs397152648	66608679	c.374−759T>C	intron 1	0.23	0.22	0.91	0.13 *	T:(C)
rs1095048831	66608524	c.374−604A>C	intron 1	0.16	0.17	0.89	0.09 *	A:(C)
rs1095048830	66608467	c.374−547A>C	intron 1	0.02	0.02	1.0	0.01	A:(C)
rs1095048829	66608461	c.374−541C>T	intron 1	0.18	0.22	0.80	0.13 *	C:(T)
rs1095048849	66606564	c.747−983T>A	intron 2	0.04	0.04	1.0	0.02	T:(A)
rs1095048848	66606554	c.747−993T>C	intron 2	0.52	0.50	0.67	0.48 *	T:(C)
rs1095048847	66606457	c.748−927A>T	intron 2	0.06	0.07	0.35	0.03	A:(T)
rs1095048846	66605717	c.748−187A>G	intron 2	-	-	-	-	A:G
rs1095048842	66605570	c.748−40delA	intron 2	-	-	-	-	A:_

SNP ID: SNP identification number; Chr position: SNP position on ECA chromosome 18; HGVS: Human Genome Variation Society; ObsH: observed heterozygosity; ExpH: expected heterozygosity; HWpv: Hardy–Weinberg test *p*-value; MAF: minor allele frequency (minor allele in brackets); *: SNPs with MAF > 0.05, submitted to association analysis with sport performance traits. SNPs rs69472472, rs1095048834, rs1095048850 and rs1095048846 were polymorphic only in two samples (KY746356 and KY746357) and they were not included in the genotyping process.

**Table 2:** Raw *p*-values and significance for sporting performance traits of the individual horse, according to genotype at equine MSTN (SNP with minimum allele and genotype frequencies higher than 0.05, one SNP at a time) and fixed effects computed in model (M1); samples from 180 Anglo-Arabian horses running 1239 races in their debut year (three year of age).

SNPs at *MSTN* (ECA18)	Traits ^1^	Genotype Effect	Fixed Effects	
			Sex	ArB ^2^
rs397152648	Total prizes won	0.047 *	0.002 **	0.002 **
	PrIndex	0.034 *	0.001 ***	0.001 ***
	1st-PlIndex	0.079	0.118	0.001 ***
	Tot-PlIndex	0.225	0.092	0.001 ***
rs1095048831	Total prizes won	0.941	0.004 **	0.008 **
	PrIndex	0.663	0.003 **	0.002 **
	1st-PlIndex	0.875	0.153	0.002 **
	Tot-PlIndex	0.804	0.133	0.001 ***
rs1095048829	Total prizes won	0.578	0.002 **	0.008 **
	PrIndex	0.493	0.002 **	0.003 **
	1st-PlIndex	0.680	0.082	0.005 **
	Tot-PlIndex	0.282	0.071	0.001 ***
rs1095048848	Total prizes won	0.736	0.005 **	0.003 **
	PrIndex	0.960	0.003 **	0.001 ***
	1st-PlIndex	0.813	0.126	0.002 **
	Tot-PlIndex	0.993	0.098	0.001 ***

^1^ PrIndex, prize index, the ratio between the total prize won and the number of races ran; 1st-PlIndex, first place index, the ratio between the number of first places and the number of races ran by that horse; Tot-PlIndex, total places index, the ratio between the number of places (sum of first, second and third places) and the number of races ran by that horse; ^2^ ArB, Arabian blood percentage; *** *p* < 0.01; ** *p* < 0.01; * *p* < 0.05; no asterisk: non-significant.

**Table 3:** Raw *p*-values and significance for sporting performance traits in the individual race, according to genotype at equine *MSTN* (SNP with minimum allele and genotype frequencies higher than 0.05, one SNP at a time) fixed effects, and the proportion of variance (in percentage) explained by the random effects of the individual horse and the jockey computed in model (M2); samples from 1239 races ran by 180 Anglo-Arabian horses in their debut year (three year of age).

SNPs at *MSTN* (ECA18)	Traits ^1^	Genotype Effect	Fixed Effects ^2^			Random Effects ^3^	
			Sex	ArB	D	H	J
rs397152648	ArIndex	0.042 *	0.171	0.001 ***	0.001 ***	25.02	3.00
	PuIndex	0.090	0.001 ***	0.001 ***	0.001 ***	11.01	3.24
	SuIndex	0.034 *	0.001 ***	0.003 **	0.001 ***	17.03	1.20
rs1095048831	ArIndex	0.978	0.243	0.002 **	0.001 ***	26.53	3.35
	PuIndex	0.682	0.003 **	0.001 ***	0.001 ***	11.48	3.35
	SuIndex	0.798	0.001 ***	0.011 *	0.001 ***	17.47	1.08
rs1095048829	ArIndex	0.485	0.141	0.005 **	0.001 ***	25.69	3.54
	PuIndex	0.276	0.001 ***	0.001 ***	0.001 ***	11.55	3.18
	SuIndex	0.330	0.001 ***	0.008 **	0.001 ***	17.05	1.28
rs1095048848	ArIndex	0.795	0.229	0.002 **	0.001 ***	25.91	3.78
	PuIndex	0.868	0.002 **	0.001 ***	0.001 ***	10.90	3.73
	SuIndex	0.865	0.001 ***	0.006 **	0.001 ***	16.85	1.64

^1^ ArIndex: arrival index, the complement to one of the ratios between the place of the individual horse at the finish line and the number of horses at the starting line of that race; PuIndex: purse index, the ratio between the prize won by the individual horse and the purse of that race; SuIndex: success index, the multiplication between the ArIndex and the prize won by the individual horse in that race; ^2^ ArB, Arabian blood percentage; D, race distance; ^3^ H, individual horse; J, jockey; *** *p* < 0.01; ** *p* < 0.01; * *p* < 0.05; no asterisk: non-significant.

**Table 4:** Least square means of sporting performance traits of the individual horse and race according to the SNPs genotypes at equine *MSTN* gene (SNP with minimum allele and genotype frequencies higher than 0.05, one SNP at a time); samples from 180 Anglo-Arabian horses running 1239 races in their debut year (three year of age).

SNPs at *MSTN* (ECA18)		rs397152648		rs1095048831		rs1095048829		rs1095048848		
Genotype		TT	TC	AA	AC	CC	CT	CC	CT	TT
Individual horse	(n)	(136)	(41)	(149)	(29)	(141)	(33)	(41)	(93)	(46)
Traits ^1^	Total prizes, €	6213	9315 *	6748	6619	7131	6183	6618	6965	5818
	PrIndex, €	742	1115 *	787	872	853	722	776	810	767
	1st-PlIndex	0.164	0.220	0.171	0.177	0.181	0.166	0.161	0.178	0.184
	Tot-PlIndex	0.378	0.442	0.388	0.374	0.404	0.341	0.389	0.382	0.385
Individual race	(n)	(943)	(278)	(1037)	(186)	(937)	(251)	(265)	(657)	(317)
Traits ^2^	ArIndex	0.477	0.545 *	0.485	0.484	0.492	0.467	0.470	0.481	0.497
	PuIndex	0.110	0.133 *	0.115	0.109	0.120	0.104	0.120	0.112	0.115
	SuIndex	719	1056	774	820	838	671	814	751	717

^1^ PrIndex, prize index, the ratio between the total prize won and the number of races ran; 1st-PlIndex, first place index, the ratio between the number of first places and the number of races ran by that horse; Tot-PlIndex, total places index, the ratio between the number of places (sum of first, second and third places) and the number of races ran by that horse; ^2^ ArIndex: arrival index, the complement to one of the ratio between the place of the individual horse at the finish line and the number of horses at the starting line of that race; PuIndex: purse index, the ratio between the prize won by the individual horse and the purse of that race; SuIndex: success index, the multiplication between the ArIndex and the prize won by the individual horse in that race; * for each SNP significant differences in genotype comparison at *p* < 0.05; no asterisk: non-significant.

## Data Availability

Data is contained within this article and Supplementary Material.

## Supplementary Materials

The following are available online at https://www.mdpi.com/article/10.3390/ani11040964/s1, Table S1: Oligonucleotides used for PCR amplification and sequencing of the entire *MSTN* gene; Table S2: Levels and total numbers of horses and races according to the phenotypic fixed effects considered in the statistical models; Figure S1: Linkage disequilibrium analysis at the *MSTN* gene in Anglo-Arabian horses (*n* = 180).
