# Supplementary material for: Polymorphisms at Myostatin Gene (MSTN) and the Associations with Sport Performances in Anglo-Arabian Racehorses
Source: Animals (Basel). 2021 Mar 30;11(4):964. doi: 10.3390/ani11040964 (PMC8065447; doi:10.3390/ani11040964)
Supplement: Supplementary file 1 [file animals-11-00964-s001.pdf]

Table S1. Oligonucleotides used for PCR amplification and sequencing of the entire *MSTN* gene. Primer pairs SHOR3737F / SHOR3737R (DNA fragment 3) and SHOR_6F / SHOR_6R (DNA fragment 6) were used for genotyping by sequencing the sample population.

Fragment	Primers′ name	Primer sequence 5′- 3′	Fragment lenght (bp)	Location of the primer	Annealing temperature (°C)
P	SHOR_PF	AACTTCTCTTTTAATACAGGTCTTCC	832	Promoter region	56
	SHOR_PR	GGCGCAGTTTACTGAGGATT		Exon 1	
1	SHOR_1F	AATTTTGCTTGGCATTGCTC	834	Promoter region	55
	SHOR_1R	GCAACCAAACGCAATTATGA		Intron 1	
2	SHOR_2F	AACAATCATTACCATGCCTACAGA	850	Exon 1	56
	SHOR_2R	TCCTCCCTCCCAAGAAGAAT		Intron 1	
3	SHOR3737F	TCAAAGAGGTTATAGCTCAGAGTCC	810	Intron 1	56
	SHOR3737R	GAGACACCGTGGAGGAACAT		Intron 1	
4	SHOR_4F	TCCCGAGGCTCAGTTAGTTC	779	Intron 1	56
	SHOR_4R	CAGGCTGTTTGAGCCAATTT		Exon 2	
5	SHOR_5F	CATCAAACCCATGAAAGACG	805	Exon 2	56
	SHOR_5R	GCATCAACAGCCTGCAAAAT		Intron 2	
6	SHOR_6F	CCCCCAGAAGAGTGTCAAAT	849	Intron 2	56
	SHOR_6R	ACGTTACTAAGTTTACGTTAAAATGCT		Intron 2	
7	SHOR_7F	TTCAGTCTTCATGTGGTCTTGG	923	Intron 2	56
	SHOR_7R	TGACTTTTCCCTATGGCTCAA		Intron 2	
8	SHOR_8F	ACCTAGGGAATGGAGGATGG	818	Intron 2	56
	SHOR_8R	AGCACCCACAGCGATCTACT		Exon 3	
9	SHOR_9F	TGCTCTGGAGAGTGTGAATTTG	846	Exon 3	56
	SHOR_9R	TGCACCTGAAGAAAGGAGAAA		3′UTR	

Table S2. Levels and total numbers of horses and races according to the phenotypic fixed effects considered in the statistical models.

Effect	levels	n° horses	n° races
Sex	male	72	478
	female	108	761
Arabian blood percent	< 50	112	712
	≥ 50	68	527
Race distance (meters)	Short (1,000 - 1,400)	-	130
	Middle (1,450 - 1,800)	-	869
	Long (1,850 - 2,400)	-	240

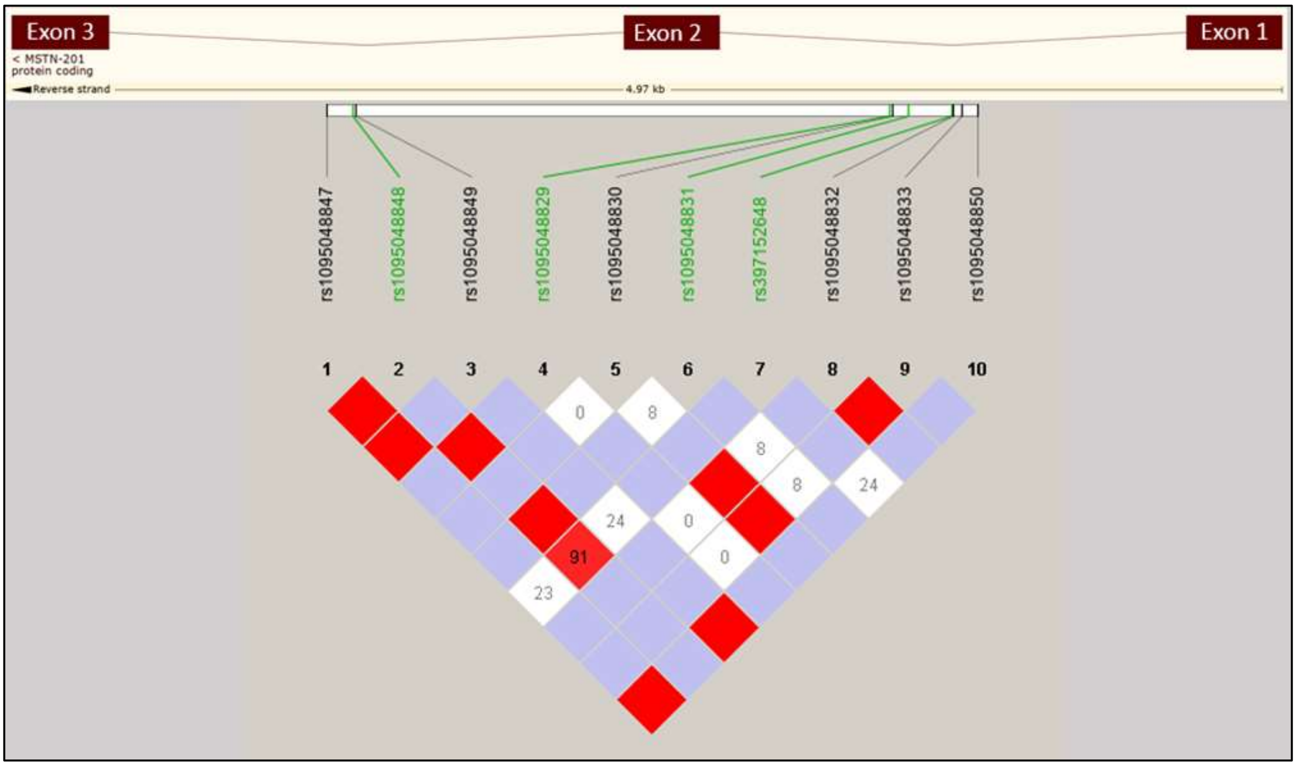

Figure S1. Linkage disequilibrium analysis at the *MSTN* gene in Anglo-Arabian horses (n = 180). SNPs are reported with their position at chromosome ECA18 (in green the SNPs showing MAF>0.05). Haploview plot of pairwise D': red, D' = 1.0 and logarithm of the odds (LOD) ≥2.0; white, D' < 1.0 and LOD <2.0. *MSTN* gene image was exported from Ensembl (http://www.ensembl.org/index.html).
